# Genomic features of meiotic crossovers in diploid potato

**DOI:** 10.1093/hr/uhad079

**Published:** 2023-04-19

**Authors:** Xiuhan Jiang, Dawei Li, Hui Du, Pei Wang, Liang Guo, Guangtao Zhu, Chunzhi Zhang

**Affiliations:** National Key Laboratory of Crop Genetic Improvement, Huazhong Agricultural University, Wuhan 430070, China; Shenzhen Branch, Guangdong Laboratory of Lingnan Modern Agriculture, Genome Analysis Laboratory of the Ministry of Agriculture and Rural Affairs, Agricultural Genomics Institute at Shenzhen, Chinese Academy of Agricultural Sciences, Shenzhen, Guangdong 518120, China; Shenzhen Branch, Guangdong Laboratory of Lingnan Modern Agriculture, Genome Analysis Laboratory of the Ministry of Agriculture and Rural Affairs, Agricultural Genomics Institute at Shenzhen, Chinese Academy of Agricultural Sciences, Shenzhen, Guangdong 518120, China; Shenzhen Branch, Guangdong Laboratory of Lingnan Modern Agriculture, Genome Analysis Laboratory of the Ministry of Agriculture and Rural Affairs, Agricultural Genomics Institute at Shenzhen, Chinese Academy of Agricultural Sciences, Shenzhen, Guangdong 518120, China; Shenzhen Branch, Guangdong Laboratory of Lingnan Modern Agriculture, Genome Analysis Laboratory of the Ministry of Agriculture and Rural Affairs, Agricultural Genomics Institute at Shenzhen, Chinese Academy of Agricultural Sciences, Shenzhen, Guangdong 518120, China; National Key Laboratory of Crop Genetic Improvement, Huazhong Agricultural University, Wuhan 430070, China; The AGISCAAS-YNNU Joint Academy of Potato Sciences, Yunnan Normal University, Kunming, Yunnan 650500, China; National Key Laboratory of Crop Genetic Improvement, Huazhong Agricultural University, Wuhan 430070, China; Shenzhen Branch, Guangdong Laboratory of Lingnan Modern Agriculture, Genome Analysis Laboratory of the Ministry of Agriculture and Rural Affairs, Agricultural Genomics Institute at Shenzhen, Chinese Academy of Agricultural Sciences, Shenzhen, Guangdong 518120, China

## Abstract

Meiotic recombination plays an important role in genome evolution and crop improvement. Potato (*Solanum tuberosum* L.) is the most important tuber crop in the world, but research about meiotic recombination in potato is limited. Here, we resequenced 2163 F_2_ clones derived from five different genetic backgrounds and identified 41 945 meiotic crossovers. Some recombination suppression in euchromatin regions was associated with large structural variants. We also detected five shared crossover hotspots. The number of crossovers in each F_2_ individual from the accession Upotato 1 varied from 9 to 27, with an average of 15.5, 78.25% of which were mapped within 5 kb of their presumed location. We show that 57.1% of the crossovers occurred in gene regions, with poly-A/T, poly-AG, AT-rich, and CCN repeats enriched in the crossover intervals. The recombination rate is positively related with gene density, SNP density, Class II transposon, and negatively related with GC density, repeat sequence density and Class I transposon. This study deepens our understanding of meiotic crossovers in potato and provides useful information for diploid potato breeding.

## Introduction

Meiosis is a specific type of cell division that occurs in sexually reproducing organisms, whereby one cell undergoes two rounds of chromosome segregation to form four ploidy-reduced daughter cells. A unique situation faced during the first meiotic division is homologous chromosome synapsis, followed by reciprocal exchange of non-sister chromatids; this latter step is termed crossover. Meiotic recombination increases the genetic diversity of a population and is an important tool used by breeders to break the linkage between genes and create new breeding material. Meiotic recombination therefore has played and still plays an important role in genome evolution and crop improvement and domestication [1, 2].

In most eukaryotes, recombination does not occur evenly across the chromosomes, and crossover preferentially takes place in narrow regions called recombination hotspots [3]. Previous studies have shown that recombination hotspots are not conserved among species and vary among species and between sexes [4], and even in different subspecies of the same species in rice (*Oryza sativa*) [5]. However, all plant species exhibit severe recombination suppression in the pericentromeric region [6]. This observation suggests that the distribution of recombination in different species or within the same species with different genetic backgrounds is different but follows conserved laws. In addition, during meiosis, when a crossover occurs at one position on a chromosome, it may suppress the chance of other recombination sites occurring nearby; this phenomenon is termed crossover interference [7]. Recombination has two sides: it can increase the genetic diversity of organisms and promote adaptation to a changing and dynamic environment, and it can also break favorable combinations of genes already present and negatively affect organisms [8–10]. Resolving the genetic recombination landscape of a population and exploring its genomic characteristics are therefore of great significance for crop improvement.

Potato is the world’s most important tuberous food crop, with about 1.3 billion people worldwide consuming it as a staple food [11]. Most cultivated potatoes are autotetraploid, with a highly heterozygous genome that complicates genetic mapping analyses. Indeed, compared to other seed crops [12–14], only a few studies have explored meiotic recombination in potato using molecular marker to construct the genetic map or by using fluorescence in situ hybridization, but information provided is limited [15–17]. With the rapid development of diploid potato breeding over the past decade, many segregating diploid populations have been developed that facilitate meiosis research in potato. Moreover, the marked and continuous drop in costs associated with high-throughput sequencing efforts has opened new avenues to our understanding of meiosis by producing high-resolution recombination maps at the whole-genome level. One study discovered that meiotic crossovers were associated with open chromatin and enriched with *Stowaway* transposons in potato [18]. However, this study only used two small diploid populations. To what extent meiotic crossovers differ among different genetic backgrounds is largely unknown. In addition, no genetic loci controlling the number of crossovers has been identified in potato.

Here, we constructed five high-resolution genetic maps of diploid potatoes by large-scale resequencing of segregating F_2_ individuals. We identified many meiotic hotspots and cold regions, as well as a quantitative trait locus (QTL) controlling recombination number in potato. The high-resolution crossover datasets presented here provide a unique opportunity to understand the crossover patterns and their genomic structural features in an important crop.

## Results

### Identification of genome-wide recombination sites in five F_2_ populations

To explore the genome-wide recombination landscape in potato, we resequenced 2163 F_1_ individuals from selfed populations of five diploid clones: Upotato 1 (*n* = 203), RH (*n* = 410), PG6359 (*n* = 542), C10–20 (*n* = 502), and D43–7 (*n* = 506). We sequenced each F_2_ plant from Upotato 1 to an average depth of 14$\times$ and those from the other four populations to a lower depth of 1.5$\times$ to 3.0$\times$. Upotato 1 is an F_1_ hybrid derived from a cross between the two highly homozygous diploid inbred lines A6–26 and E4–63 [19]. We identified 3 161 560 single nucleotide polymorphisms (SNPs) between the two parents with which to genotype the F_2_ progeny (Table 1; Fig. S1, see online supplementary material). As the parents of the other four clones are not clear, we used the pipeline of parent-independent genotyping we previously developed to distinguish between the two haplotypes [20]. The number of SNPs used for genotyping varied from 919 012 to 1 336 924 depending on the population (Table 1; Fig. S1, see online supplementary material). The high-density SNPs guaranteed the accuracy and relative precision of recombination site detection.

**Table 1:** Summary of bin maps for the five F_2_ populations

	**Upotato 1**	**RH**	**PG6359**	**C10–20**	**D43–7**
No. of F_2_ individuals	203	410	542	502	506
No. of phased SNPs	3 161 560	1 262 859	1 020 160	1 336 924	919 012
No. of bins	2514	1875	1994	2132	1595
No. of bins with SDs^a^	463	653	665	788	551
Genomic regions with SDs (Mb)	198	234	203	221	225
Genomic regions with SDs (%)	27.1	32.0	27.8	30.2	30.8

a χ^2^ test, *P* < 0.001. SD, segregation distortion.

**Figure 1:**
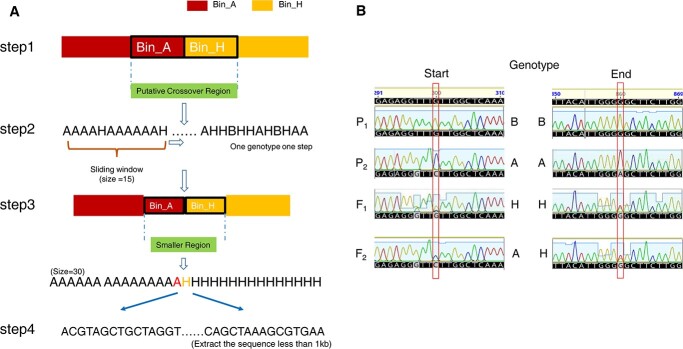
Identification and validation of crossovers. **A** Crossover identification. **B** Validation of crossover: P_1_(B), P_2_(A), F_1_(H), F_2start_(A), and F_2end_(H). P_1_ represents one parent, P_2_ represents the other parent, F_1_ is the cross of two parents, and F_2_ is the self progeny of F_1_.

Since the F_2_ population of Upotato 1 was deeply sequenced, we developed a pipeline to identify crossover intervals (Fig. 1A). First, we used a sliding window (size = 1 Mb, step = 100 kb) to genotype all individuals and used the middle position of two adjacent windows exhibiting a change in genotype as the hypothetical recombination break point. We then set two hypothetical bins, designated Bin_A and Bin_H, on either side of each putative recombination break point. Then, we extracted the sequences between the two bins (step 1). We performed the same analysis with a smaller sliding window (size = 15 SNPs, step = 1 SNP) to continue genotyping (step 2) and extracted the genotypes of two adjacent windows where the genotype changed, culminating with the identification of a crossover interval between the two SNPs separated by the smallest distance (step 3). To verify the accuracy of our method (Fig. 1B), we randomly selected 20 putative crossovers (resolved to less than 1 kb) and amplified the target sequences (Table S1, see online supplementary material) for Sanger sequencing (step 4). We confirmed the presence of a crossover in all sequenced PCR products, thus validating our pipeline. For the other four populations, as the low sequencing coverage limited the accurate identification of heterozygous SNPs, we directly used the larger sliding window approach (size = 1 Mb, step = 100 kb) to identify putative recombination sites. With these five datasets of crossover positions, we reconstructed the bin maps for each of the five populations and estimated segregation distortion across the genome (Table 1; Fig. S2, see online supplementary material). We determined that Upotato 1 has the smallest regions affected by segregation distortion, indicating that we effectively eliminated some large-effect deleterious mutations through previous breeding by genomic selection. We found that the segregation distortions were different among five populations, which was consistent with previous studies [21, 22].

### Comparison of crossover distribution among different populations

**Figure 2:**
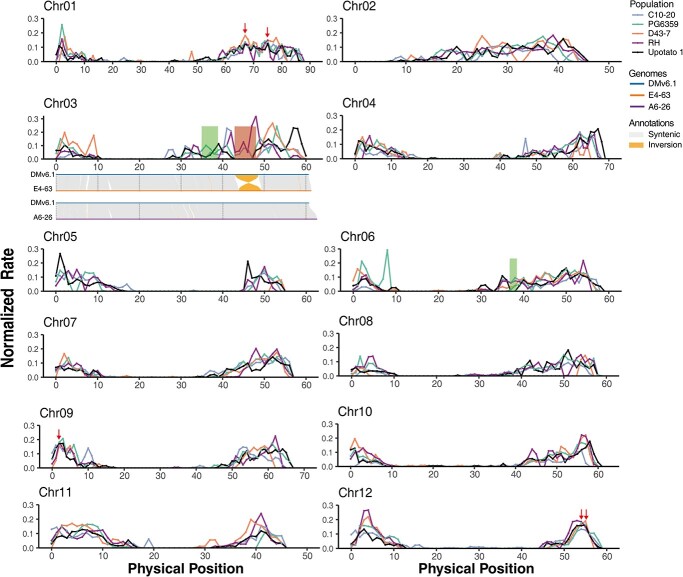
Distributions of crossovers among different groups. The x-axis represents the physical location along the genome in Mb. The y-axis represents normalized recombination rate (number of recombination events/total number of individuals). The synteny graph below chromosome 3 is the structural genomic variants between A6–26 (female of Upotato 1) and E4–63 (male of Upotato 1) with DMv6.1. The red shading represents regions with suppressed recombination. The green shading represents false positive regions. Red arrows point to shared recombination hotspots.

To compare the recombination frequency among different populations, we counted the number of recombination events using a sliding window of 2 Mb in size with a step size of 1 Mb (Fig. 2). We noticed a severe suppression of recombination near the centromeric regions, where we detected almost no crossover, as in previous studies [6, 23, 24]. Furthermore, we calculated the genome-wide recombination rate of the five populations and identified 301 crossover hotspots across the five populations, using a threshold of 12 cM/2 Mb, which is about three times the genome-wide recombination rate in these populations. We observed five shared hotspots mapping to chromosomes 1, 9, and 12.

In addition, we observed regions with suppressed recombination in euchromatin, such as the long arm of chromosome 3 in two populations (Upotato 1 and PG6359). We also identified some regions that are likely not subjected to recombination suppression but are false positives due to their low SNP density (green shading in Fig. 2; Fig. S1, see online supplementary material). The availability of high-quality biparental genomes for Upotato 1 allowed us to explore the effect of structural variation between genomes on crossover distribution [19]. Accordingly, we analysed structural variants between the two parents of Upotato1 (A6–26 and E4–63) by comparing their genomes to the potato reference genome DMv6.1 and detected an inversion in E4–63 on chromosome 3. Notably, their positions coincided with those from regions with recombination suppression. The recombination suppression region on chromosome 3 colocalized with the yellow tuber flesh locus, which is an important breeding target. We employed the same method with PG6359 [25] and obtained largely congruent results between the positions of inversions and where recombination suppression occurred (Fig. S3, see online supplementary material). Our findings confirm that, as a clonally propagated crop, the structural variants hidden in the potato genome affect meiotic recombination, which is crucial for breeding decisions.

### Genomic features of crossovers

**Figure 3:**
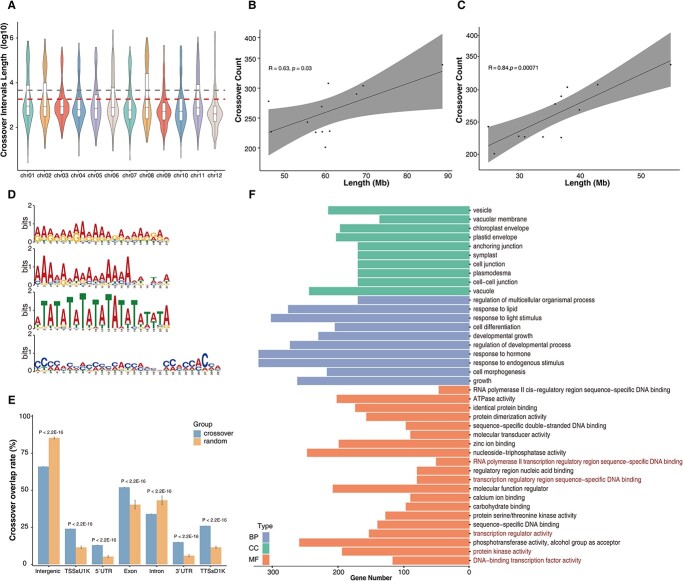
Crossover intervals and downstream analysis. **A** Distribution of crossover interval sizes for each chromosome. The horizontal red dashed line represents crossover intervals equal to 2 kb, and the gray dashed line represents crossover intervals equal to 5 kb. All values were Log_10_-normalized. **B** and **C** Correlation of crossover number with chromosome length (**B**) and euchromatin length only (**C**). **D** Sequence logos of conserved sequences within crossover intervals. **E** Overlap rate of crossovers with genomic features. **F** Gene Ontology term enrichment analysis of genes associated with crossovers. BP, biological process; CC, cellular component; MF, molecular function.

Deep sequencing of the F_2_ population from Upotato 1 facilitated the delineation of each crossover interval to two adjacent SNPs, resulting in a median interval size of 728.5 bp (*n* = 3140). Most (70.2% or 2205) crossover intervals were less than 2 kb, and 78.25% were less than 5 kb (Fig. 3A). The recombination rate of chromosome 2 of 5 cM/2 Mb was the highest in this population, with an average crossover interval size of 930 bp, while chromosome 12 showed the lowest recombination rate at 2.84 cM/2 Mb. In Arabidopsis, the number of crossovers is positively associated with chromosome length [26]. Therefore, we performed a correlation analysis between the number of crossovers and chromosome length and euchromatin length using the data from the Upotato1 F_2_ population and reached a similar conclusion as in Arabidopsis for chromosome length (R = 0.63, *P* = 0.03) and euchromatin length (R = 0.84, *P* = 0.00071; Fig. 3B and C). We extracted all crossover intervals less than 5 kb in size as a high-resolution dataset (*n* = 2457) (Fig. 3A; Table S2, see online supplementary material) to help us detect conserved motifs using MEME suite [27]. We identified four conserved motifs in these sequences: poly-A/T, poly-AG, AT-rich, and CCN repeats (Fig. 3D), which were consistent with previous studies in tomato (*Solanum lycopersicum* L.) and Arabidopsis [28, 29]. However, we did not detect other motifs that have been reported in Arabidopsis crossover intervals [12, 29], such as CTT and CT repeats.

In addition, based on the annotation of the potato reference genome DMv6.1, we explored the relationship between high-resolution crossovers and genomic features. We observed that 57.1% (1403/2457) of all crossovers overlap with a genic region (exon or intron), with 66.4% (1631/2457) of crossovers mapping to intergenic sequences, and 23.5% (577/2457) crossovers overlapping with both gene and intergenic sequences (Fig. S4; Table S3, see online supplementary material), which is similar with a previous study in soybean [30] and two F_2_ populations of potatoes [18]. To assess whether these overlaps were random, we performed 10 000 Monte Carlo simulations on 2457 randomly selected sequences of equal length across the genome corresponding to hypothetical crossovers and calculated the random overlap rate with genomic features. For gene and intergenic, the average overlapping rates were 33.3% (±1.6%, standard deviation (SD)) for genic regions and 85.3% (±3.1%, SD) for intergenic regions, indicating that the overlap rate of our high-resolution crossover datasets is higher (genic) and lower (intergenic) than that of the random dataset (*P* < 0.001). Using the same approach for other gene structures, we detected an enrichment for crossovers in TSSsU1K (1 kb upstream of the transcription start site); untranslated regions (3′ UTRs), exons, and TTSsD1K (1 kb downstream from the transcription termination site), but they appeared to be relatively depleted from introns (Fig. 3E).

In humans and fungi, crossover hotspots are usually delimited to ~1- to 2-kb regions [31, 32]. We wished to narrow down recombination hotspots to smaller regions based on our mean crossover interval size of 728.5 bp. We used a smaller window (size = 50 kb) to calculate recombination rates for the Upotato 1 F_2_ population with its extremely high SNP density and identified 12 recombination hotspots using a recombination threshold of 2.4 cM/50 kb which is 29 times the whole-genome recombination rate of 0.084 cM/50 kb. These hotspots were all included in the hotspots already identified when using the large window (Fig. 2; Fig. S5A; see online supplementary material). We extracted the genes within the recombination hotspots for Gene Ontology (GO) enrichment analysis. These genes were enriched in pathways related to environmental stimulus regulation, such as ‘regulation of response to stimulus’, ‘response to acid chemical’, ‘response to water’, ‘protein kinase activity’, and ‘signaling receptor activity’ (Fig. S5B). These GO terms are similar to those from previous studies showing that genes related to environmental stimuli display higher recombination rates [33]. Furthermore, we also investigated the functional annotation of genes or their TSSsU1K that overlapped with a crossover. We determined that these genes (genes + TSSsU1K) are enriched for regulatory activities such as ‘transcription regulator activity’, ‘transcription regulatory region sequence specific DNA binding’, and ‘DNA binding transcription factor activity’ (Fig. 3F; Fig. S5C, see online supplementary material). These results suggest that genes harboring crossovers may play an important role in gene regulatory functions.

**Figure 4:**
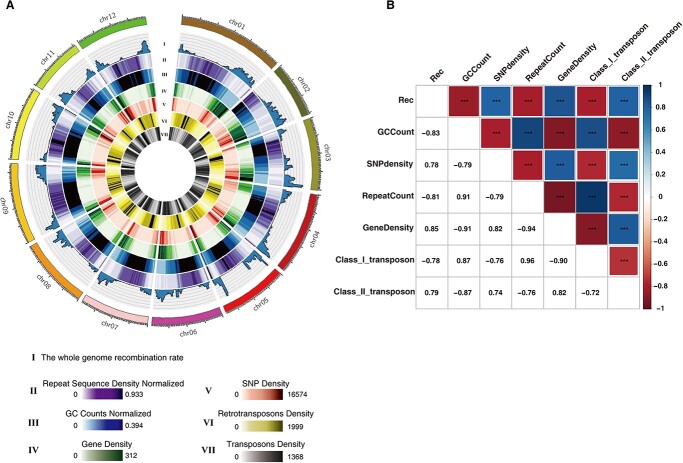
Relationship between genomic features and crossovers. **A** Circos plot of different genomic features and crossovers. I: The whole genome recombination rate. II: Normalized repeat sequence density (repeat sequence length/ 2 M). III: Normalized GC count (the number of G and C/2 M). IV: Gene Density V: SNP Density. VI: Retrotransposons Density VII: Transposons Density. All were calculated using a sliding window (size = 2 Mb, step = 1 Mb). **B** Heatmap showing the extent of correlation between recombination rate and genomic features.

Next, we explored the distribution of crossovers in relation to chromosome GC content, repeat sequence density, gene density, SNP density, class I transposon density, and class II transposon density (Fig. 4A). We detected a significant and positive correlation between the distribution of crossovers and SNP density, gene density, and class II transposon density but a negative correlation with GC content, repeat density, and class I transposon density (Fig. 4B), all of which correspond to well-known genomic features associated with plants [34, 35]. The pericentromeric regions of chromosomes are highly heterogeneous, with highly condensed heterochromatin that is rich in repeat sequences. Transcription is more difficult to complete in such tightly compressed chromatin, and crossover formation is more difficult. Therefore, crossover occurs preferentially in euchromatic regions with a low repeat density and high gene content.

### QTL analysis of crossover counts

**Figure 5:**
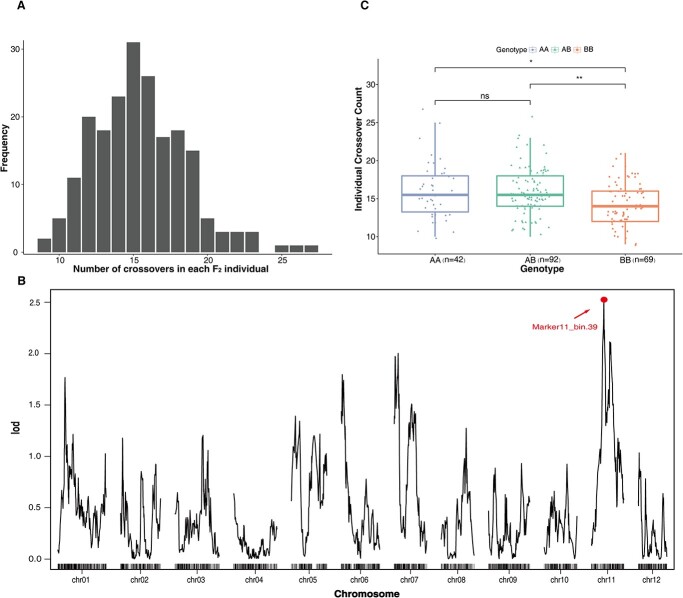
QTL mapping of crossover number in the F_2_ population of Upotato 1. **A** Distribution of the number of crossovers in each individual. **B** QTL mapping of crossover number. The highest peak is marked with a red circle, and the marker with the highest LOD value was named *Marker11_bin.39.***C** Distribution of crossover numbers among F_2_ individuals with different genotypes at *Marker11_bin.39*.

To test whether loci might regulate the recombination rate in potato, we performed a genetic analysis using the F_2_ population from Upotato 1. Most chromosomes had crossover numbers of 1 to 2 (Table S4, see online supplementary material), with few examples with four or five crossovers (<1.5%), which indicates the existence of crossover interference. Besides, some chromosomes in certain individuals had no crossover occurrence (Table S4, see online supplementary material), which is a common phenomenon in potato [18, 20]. It may depend on the pattern of strand repair following DNA double strand break [36]. The number of crossovers in each F_2_ plant ranged from 9 to 27, with an average of 15.5, close to a normal distribution (Fig. 5A). We performed QTL mapping using the number of crossovers per individual as phenotypic data (Fig. 5B). We detected one peak on chromosome 11 (logarithm of the odds (LOD) = 2.53) that explains 5.6% of the standing phenotypic variance. We calculated the number of crossovers for the three possible genotypes (AA, AB, and BB) at this QTL (Fig. 5C). The AA and AB genotypes showed no difference in their crossover numbers, while the BB genotype had 12% fewer crossovers than AA, indicating that the A allele (A6–26 corresponds to the A allele) is a dominant QTL regulating the recombination rate in diploid potato.

## Discussion

Meiotic recombination is an important resource for plant genome evolution and crop genetic improvement [37]. Recombination is not randomly distributed along the genome, and the distribution of recombination differs among species or materials with different genetic backgrounds [38]. Compared to a previous study [18], we used more materials including different genetic backgrounds to study the meiotic recombination in potato. We confirmed that recombination distribution differed among materials in diploid potato and identified five shared recombination hotspots. GO enrichment analysis of the genes located in the shared hotpots showed that they were enriched in functions related to adaptation to the environment and responses to external stimuli (Fig. S6, see online supplementary material), suggesting that the genes that reside in the crossover hotspots may play an important role in potato survival and evolution. Besides, we also detected several examples of regions with suppressed recombination that were associated with large chromosome structural rearrangements (Fig. 2). A previous study in *Neurospora tetrasperma* showed that suppression of sex chromosome recombination is not associated with structural variation. This finding suggests that other factors also contribute to recombination suppression, especially specific methylation modifications [39]. Heterochromatin is characterized by many repetitive sequences and is highly constricted for double stand break (DSB) to occur. By contrast, euchromatin regions show much higher chromatin accessibility and high transcriptional activity; these regions are more amenable to DSB and recombination. The contrasting crossover frequencies of heterochromatin and euchromatin thus exhibit severe recombination suppression of heterochromatin reorganization and result in an uneven crossover distribution [38].

Consensus motif analysis identified several sequence motifs that occurred frequently in diploid potato at crossovers: poly-AG, poly-A/T, CCN repeats, and the relatively weakly frequent poly-AT. Poly-A/T tracks were found repeatedly in the binding site of an Arabidopsis auxin response factors in a previous study [40]; this type of sequence is also an important recombination target region in yeast (*Saccharomyces cerevisiae*) [32]. CCN repeats were previously shown to be associated with recombination in Arabidopsis genes and linked to elevated chromatin accessibility [41]. Furthermore, AT-rich motifs have been reported to be involved in the formation of recombination hotspots and DSB in Arabidopsis [42]. For the genomic features associated with crossovers, we established that crossovers preferentially occur within genes rather than in intergenic regions. We speculate that conventional crossovers do not affect the expression of certain genes, but crossovers that lead to gene conversion can result in changes in gene function by creating point mutations in gene coding regions, which may be a means for plants to adapt to environmental changes by selection. This notion would be in agreement with previous studies that identified many genes related to environmental adaptation with high recombination rates [33]. This idea is also supported by our results on the enrichment of recombination hotspot genes.

Previous studies have confirmed that crossover number and distribution are regulated and influenced by many genes and proteins and can be affected by environmental factors [2, 37]. It is important to investigate the influence of such genetic factors on variation of recombination rate. We performed a scan for QTLs using individual crossover numbers as phenotypes and localized one QTL on chromosome 11. Related research has been performed on other plants, as well as some animals [43, 44]. The QTL scan also suggests that meiotic recombination is a polygenic trait, consistent with previous reports. In barley (*Hordeum vulgare*), 16 QTLs were identified when using a genetic map generated from 45 segregating populations derived from crosses between 23 spring barley accessions [45]. One possible explanation for the low number of detected QTLs in this study is that the population used here did not show sufficient variation in meiotic recombination. It may be possible to raise the number of crossovers by growing plants at a high temperature or in other extreme environments and constructing new populations to detect more QTLs for recombination. Up to now, some genes affecting recombination rate have been cloned, such as *Atprd1* in Arabidopsis [46], *Ph1* in Wheat [47] (*Triticum aestivum*), *Phs1* in maize [48] (*Zea mays L.*), *Pair1* in rice [49]. Recently, one study in potato reported that the *DMC1* gene is important in recombination [50]. However, none of these genes were located on chromosome 11, indicating there may be some other genes affecting the recombination in our population.

In conclusion, we compared the recombination landscape of five different populations, identified five common recombination hotspots, analysed the genomic features of the crossover regions using higher-resolution crossover datasets, and identified a QTL controlling the number of crossovers. The use of tetrad spore and pollen sequencing combined with methylation data will help us explore the mechanisms underlying meiotic recombination more clearly. Our results provide useful information for the diploid potato breeding.

## Materials and methods

### Plant materials, DNA isolation, and library preparation

A total of 2163 F_2_ plants derived from five diploid potato accessions, RH, PG6359 (CIP705468), C10–20, D43–7, and Upotato 1, were used for genetic analysis in this study. RH and PG6359 are self-compatible diploid clones that were previously reported [20]. C10–20 and D43–7 are two self-compatible BC_1_ clones. C10–20 is derived from a cross between the diploid landrace CIP701165 and E172 (containing the *S-locus inhibitor* gene, *Sli*) and then backcrossed to CIP701165, and D43–7 is derived from a cross between the diploid landrace CIP703280 and E172 and then backcrossed to CIP703280. PG6359 (CIP705468), CIP701165, and CIP703280 were kindly provided by International Potato Center. Upotato 1 is a F_1_ hybrid, derived from the cross of two homozygous inbred lines A6–26 and E4–63 [19].

Genomic DNA was isolated using a modified cetyltrimethylammonium bromide method [51] and subjected to whole-genome resequencing using Illumina HiSeq2000/2500 (San Diego, California, USA) platforms or a BGI MGISEQ-T7 (Shenzhen, Guangzhou, China) platform. The insert size of paired-end sequencing libraries was 250–300 bp, and the read length was 150 bp. For Upotato 1, ~10-Gb clean data were generated for each F_2_ progeny, and about 2 Gb for each F_2_ individual from the other four populations (RH, C10–20, PG6359, and D43–7). In addition, about 10 Gb of clean data was generated for each parental clone.

### SNP calling and genotyping

All population resequencing data were mapped to the potato reference genome DMv6.1 [52] with BWA [53] using default parameters. SAMtools software was used to convert mapping results into BAM format [54]; duplicated reads were marked using the Picard package in GATK [55]. BCFtools software was used for SNP detection with default parameters [56]. Filtering parameters (MQ ≥ 40, QUAL ≥40, 5 ≤ Depth ≤ 25) used Python scripts for all parental clones. For Upotato 1, the sites were extracted with genotyping of 0/0 or 1/1 in the two parental clones, while the heterozygous sites in Upotato 1 were used to define useful SNPs distinguishing the two parents. Two sets of phased SNP data were finally obtained for F_2_ genotyping. For the remaining four populations, haplotype differentiation and genotyping were performed using a previously described method [20].

### Crossover validation by sanger sequencing

To validate the accuracy of recombination breakpoints, 20 crossover intervals were randomly selected (<1 kb in size), and primers were designed upstream and downstream of the breakpoints using primer5, with a product size of about 1 kb. The PCR amplicons from the two homozygous parental lines (A6–26 and E4–63) and F_1_ (Upotato 1) and F_2_ progeny were subjected to Sanger sequencing. All sequencing results were analysed using Geneious software.

### Calculation of recombination rate and identification of hotspots

The mean genome-wide recombination rate was obtained by summing the values within all windows and dividing them by the total number of windows. The mean rate of the five population is 3.58 cM/2 Mb (Upotato 1), 3.71 cM/2 Mb (RH), 3.78 cM/2 Mb (PG6359), 3.06 cM/2 Mb (C10–20), and 3.58 cM/2 Mb (D43–7). A value of 12 cM/2 Mb was defined as the hotspot threshold and was 3.35-, 3.23-, 3.17-, 3.92-, and 3.35-fold higher than the mean genome-wide recombination rate of Upotato 1, RH, PG6359, C10–20, and D43–7, respectively. Genome structural variants were detected with minimap2 [57] and SyRI [58]. The calculation of the genome-wide recombination rate followed the formula:

$$\begin{align*}&(1) GenomeRcRate={\frac{\sum WinCoCounts}{WinCounts{\ast }N}}{\ast }100\\ &(2) WinRcRate={\frac{WinCoCounts}{N}}{\ast }100 \end{align*}$$

where *N is the number of individuals.

### Genomic features of crossovers

The BED file with information on 5′ UTR, 3′ UTR, exon, intron, TSSsU1K, TTSsD1K, and mRNA (gene) features was extracted from the annotation file of the potato reference genome DMv6.1 using a Python script, and the intergenic BED file was extracted using BEDtools (v2.29.2) [59] Sequences were randomly extracted from the whole genome of equal length as the high-resolution crossover dataset (*n* = 2457) and used to perform 10 000 Monte Carlo simulations; the average value was calculated and taken as the final result. All statistical tests were performed in R (4.1.3). The annotation of genomic transposable elements was performed using EDTA with default parameters [60]. All high-resolution crossover sequences were extracted using Seqtk (https://github.com/lh3/seqtk). MEME suite [27] was used to find conserved motifs with default parameters. The web tool jvenn [61] was used to visualize the intersection.

### Gene ontology enrichment and QTL analysis

All genes or their TSSsU1K overlapping with crossovers were used to perform a GO term enrichment analysis using clusterProfiler [62]. The potato gene GO annotation set was retrieved using AnnotationHub (https://bioconductor.org/packages/AnnotationHub/). For QTL analysis, the crossover number of each F_2_ individual was used as the phenotype. Genetic distances of individual bins were calculated using MSTmap [63]. QTL scans were performed using the usual steps of the R package qtl [64].

## Supplementary Material

Web_Material_uhad079

## Data Availability

All resequencing data have been deposited in the NCBI Sequence Read Archive under BioProject accession number PRJNA904517 (https://dataview.ncbi.nlm.nih.gov/object/PRJNA904517?reviewer=1eipbbj3d6qjbjktigm8l908h6).
