# Supplementary material for: Genomic features of meiotic crossovers in diploid potato
Source: Hortic Res. 2023 Apr 19;10(6):uhad079. doi: 10.1093/hr/uhad079 (PMC10261879; doi:10.1093/hr/uhad079)
Supplement: Web_Material_uhad079 [file web_material_uhad079.zip › Supplementary figures.docx]

**Figure S1.** Whole genome phased SNP density of five populations. The vertical direction represents 12 chromosomes, and the horizontal direction is the density of SNPs in 1-Mb window. **(A)** Upotato 1; **(B)** RH; **(C)** PG6359; **(D)** C10-20; **(E)** D43-7.


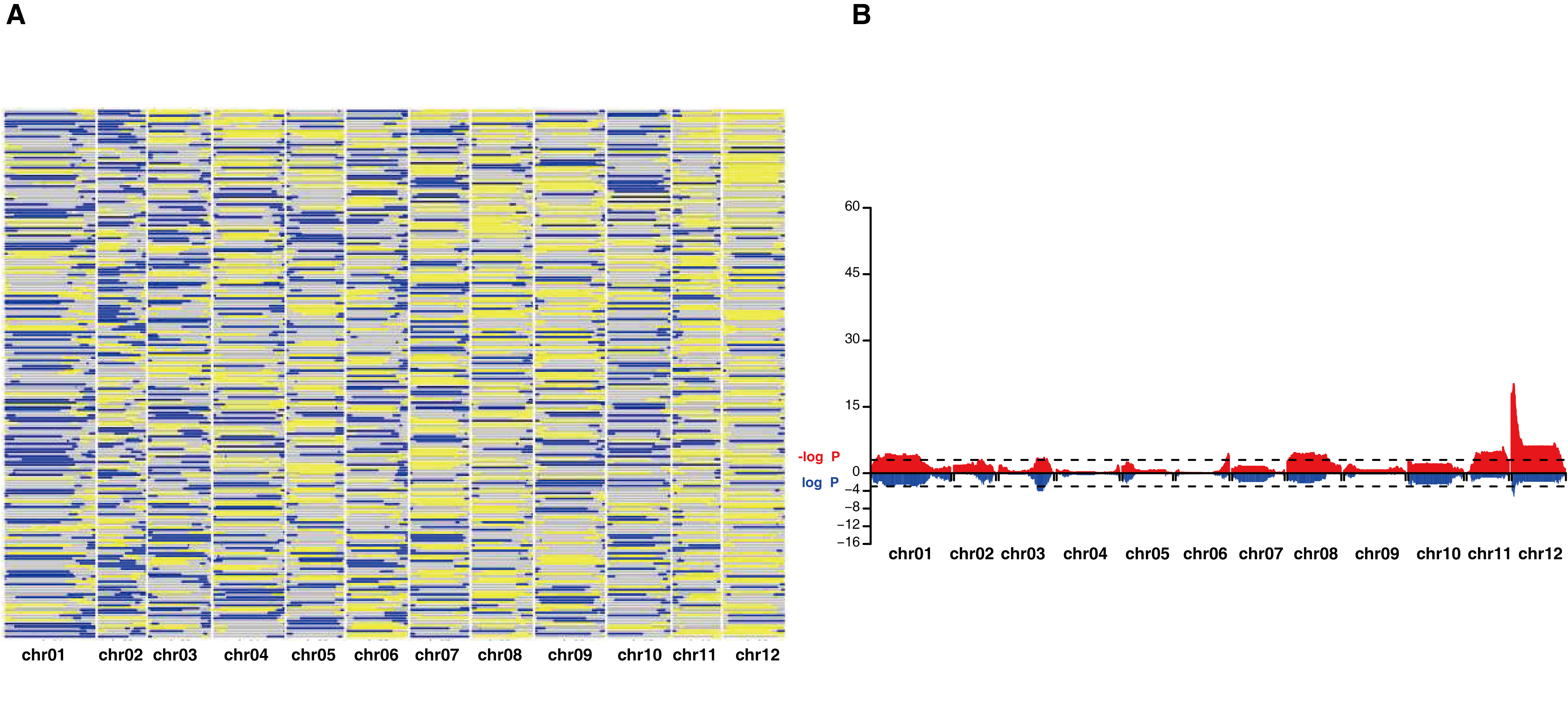


**Figure S2.** The bin map of Upotato F_2_ and the segregation distortion analysis. **(A)** The bin map of F_2_ plants from Upotato1. The blue represents the E4-63 genotype, yellow represents the A6-26 genotype, and grey represents the heterozygosity. **(B)** The segregation distortion analysis. The y-axis indicates the chi-square test value (-log_10_P), red represents the zygote, blue represents gametocytes.

**

Figure S3.** The structural variation of PG6359 genome with DMv6.1. The left figure represents the haplotype 1, and the right figure represents the haplotype 2.


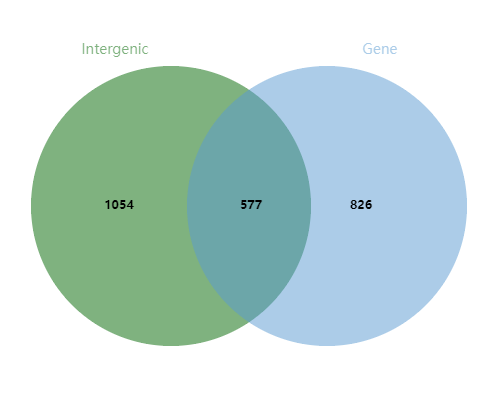


**Figure S4.** The venn graph of crossovers which overlapped with intergenic and gene regions. 1880 crossovers were specific and 577 were overlapped to both genic and intergenic regions.

**

**

**Figure S5.** Crossover hotspot of Upotato1 F_2_ and GO enrichment analysis of the genes located in crossover hotspots or with crossover occurring in TSSsU1K. **(A)** The 12 crossover hotspots in the F_2_ population of Upotato 1_._ Red dots represent the crossover hotspots. **(B)** The GO enrichment analysis of genes located in crossover hotspots. **(C)** The GO enrichment analysis of genes whose TSSsU1K overlapped the crossover.





**Figure S6.** The GO enrichment analysis of the genes located in five shared hotspots. One figure represents one shared hotspot. The size of dot represents the gene counts. The color of dot represents the rank of the P-value. **(A)** The first hotspot on chr01. **(B)** The second hotspot on chr01. **(C)** The hotspot on chr09. **(D)** The first hotspot on chr12. **(E)** The second hotspot on chr12.
