# Supplementary material for: Genomic features of meiotic crossovers in diploid potato
Source: Hortic Res. 2023 Apr 19;10(6):uhad079. doi: 10.1093/hr/uhad079 (PMC10261879; doi:10.1093/hr/uhad079)
Supplement: Web_Material_uhad079 [file web_material_uhad079.zip › Supplementary tables.docx]

| **Table S1:** PCR primers of crossovers for Sanger sequencing | | | | | | | |
| --- | --- | --- | --- | --- | --- | --- | --- |
| **Individual ID** | **Chromosome** | **Crossover interval length** | **Crossover start** | **Crossover end** | **5'-3'** | **Direction** | **Validation** |
| AxE-F2-18 | chr01 | 382 | 78034923 | 78035305 | GAGAAGAGAGTTTTAAGCTGAGATG | forward | passed |
| AxE-F2-18 | chr01 | 382 | 78034923 | 78035305 | GGATGAGGAATACGAGCCAA | reverse | passed |
| AxE-F2-62 | chr01 | 537 | 8709966 | 8710503 | AAGCGAGCACCTTACAACCC | forward | passed |
| AxE-F2-62 | chr01 | 537 | 8709966 | 8710503 | ATACATTATCTTCACTGACCTCC | reverse | passed |
| AxE-F2-205 | chr02 | 256 | 31036436 | 31036692 | CTAGTTGGGATATCACATAAGAGG | forward | passed |
| AxE-F2-205 | chr02 | 256 | 31036436 | 31036692 | GCTTGTACTTTGGTAGTGATGCC | reverse | passed |
| AxE-F2-33 | chr02 | 946 | 39190725 | 39191671 | GGATTTCTATGTTGTATTGGAGCGC | forward | passed |
| AxE-F2-33 | chr02 | 946 | 39190725 | 39191671 | CTTAATTTCAGCAACACTCCTACC | reverse | passed |
| AxE-F2-14 | chr03 | 505 | 58475722 | 58476227 | ACATAAATTACTACGATGGAGGGAG | forward | passed |
| AxE-F2-14 | chr03 | 505 | 58475722 | 58476227 | GAGACGGATTGCATTTATCGTAC | reverse | passed |
| AxE-F2-98 | chr03 | 255 | 50990077 | 50990332 | CCTGGACTCTAACTAATACCTCTAAG | forward | passed |
| AxE-F2-98 | chr03 | 255 | 50990077 | 50990332 | CCGGTATTTCCTGTAGTCCTG | reverse | passed |
| AxE-F2-99 | chr03 | 478 | 54892536 | 54893014 | GGTTTTGGTAGGATTATAGGGATTC | forward | passed |
| AxE-F2-99 | chr03 | 478 | 54892536 | 54893014 | CAAATCACAGTGGAGGAAAAAAAGG | reverse | passed |
| AxE-F2-209 | chr04 | 533 | 6053719 | 6054252 | CTATTATTTCCGTACCGACAGATTG | forward | passed |
| AxE-F2-209 | chr04 | 533 | 6053719 | 6054252 | CATCCTCCCTTTTTCCTTTGAAC | reverse | passed |
| AxE-F2-64 | chr04 | 979 | 49967519 | 49968498 | CAGTTTAAAGGAGGGAATGTTGAC | forward | passed |
| AxE-F2-64 | chr04 | 979 | 49967519 | 49968498 | GCTACTGCCACTTCCAACCC | reverse | passed |
| AxE-F2-104 | chr06 | 613 | 3743616 | 3744229 | CGATCAACCGTCTTCTTAGTAGTG | forward | passed |
| AxE-F2-104 | chr06 | 613 | 3743616 | 3744229 | CACTATACCAAGTACGCTATTTG | reverse | passed |

| **Continued** | | | | | | | |
| --- | --- | --- | --- | --- | --- | --- | --- |
| **Individual ID** | **Chromosome** | **Crossover interval length** | **Crossover start** | **Crossover end** | **5'-3'** | **Direction** | **Validation** |
| AxE-F2-10 | chr07 | 523 | 54967540 | 54968063 | GCTCTGCCACCTAGAAATGTG | forward | passed |
| AxE-F2-10 | chr07 | 523 | 54967540 | 54968063 | GAGGATGGAGCACAGTCTTCG | reverse | passed |
| AxE-F2-171 | chr08 | 218 | 50631417 | 50631635 | GAGGTTAGATTGGTCATTTCTCAT | forward | passed |
| AxE-F2-171 | chr08 | 218 | 50631417 | 50631635 | GCGTGTTTAGATAGCTTGAGTTTTCC | reverse | passed |
| AxE-F2-83 | chr08 | 308 | 38125818 | 38126126 | GTAAAGTATTCCCCTCACCGTT | forward | passed |
| AxE-F2-83 | chr08 | 308 | 38125818 | 38126126 | GCAACCTTGTCCTTCACTTCC | reverse | passed |
| AxE-F2-114 | chr09 | 566 | 57752614 | 57753180 | CGACAAACCACATACGTACTTT | forward | passed |
| AxE-F2-114 | chr09 | 566 | 57752614 | 57753180 | TGGCTTGACATTGCACTTCAG | reverse | passed |
| AxE-F2-180 | chr09 | 281 | 3743400 | 3743681 | TCCCACCTCAATTTTCCCAAA | forward | passed |
| AxE-F2-180 | chr09 | 281 | 3743400 | 3743681 | CATAAAACTAATTGGTGCAGTGGAG | reverse | passed |
| AxE-F2-204 | chr10 | 435 | 56052738 | 56053173 | GGGAGTGATGGCAAATCTTTAATC | forward | passed |
| AxE-F2-204 | chr10 | 435 | 56052738 | 56053173 | CCATTTGCTTCATTGCTTCTCTG | reverse | passed |
| AxE-F2-82 | chr10 | 193 | 56315170 | 56315363 | CACTTCGTTTACACCCTCCTCT | forward | passed |
| AxE-F2-82 | chr10 | 193 | 56315170 | 56315363 | CTGCAGCTATTTGAAGAACAGTTG | reverse | passed |
| AxE-F2-159 | chr10 | 260 | 56468604 | 56468864 | GCTACAAGGGAAAATACGCCGG | forward | passed |
| AxE-F2-159 | chr10 | 260 | 56468604 | 56468864 | CAACACTGTAGTTTTGCGTG | reverse | passed |
| AxE-F2-2 | chr11 | 213 | 2458039 | 2458252 | GGTTGTGGTTCTTCTTCTTCAATC | forward | passed |
| AxE-F2-2 | chr11 | 213 | 2458039 | 2458252 | CAGTGTTTCATGCCGTTCAAC | reverse | passed |
| AxE-F2-95 | chr12 | 160 | 54999630 | 54999790 | CCTCCGCTCTCATCTTCTTGA | forward | passed |
| AxE-F2-95 | chr12 | 160 | 54999630 | 54999790 | GATAACCTGACAGCTCTCGATAG | reverse | passed |

| **Table S2:** The summary information of crossover intervals and crossover counts | | | | | | | | |
| --- | --- | --- | --- | --- | --- | --- | --- | --- |
| **Chromosome** | **Crossover counts** | **Crossover counts per individual** | **Crossover counts (intervals less than 2 kb)** | **Crossover counts ratio (intervals less than 2 kb)** | **Crossover counts (intervals less than 5 kb)** | **Crossover counts ratio (intervals less than 5 kb)** | **Median crossover intervals (bp)** | **Median recombination rate (cM/2 Mb)** |
| chr01 | 339 | 1.67 | 216 | 63.72% | 242 | 71.39% | 990 | 3.23 |
| chr02 | 278 | 1.37 | 179 | 64.39% | 202 | 72.66% | 939 | 5.01 |
| chr03 | 308 | 1.52 | 228 | 74.03% | 258 | 83.77% | 931 | 4.25 |
| chr04 | 304 | 1.50 | 218 | 71.71% | 246 | 80.92% | 708.5 | 3.70 |
| chr05 | 243 | 1.20 | 171 | 70.37% | 194 | 79.84% | 794 | 3.59 |
| chr06 | 269 | 1.33 | 176 | 65.43% | 198 | 73.61% | 858 | 3.90 |
| chr07 | 226 | 1.11 | 169 | 74.78% | 184 | 81.42% | 668.5 | 3.26 |
| chr08 | 227 | 1.12 | 139 | 61.23% | 151 | 66.52% | 868 | 3.03 |
| chr09 | 290 | 1.43 | 230 | 79.31% | 250 | 86.21% | 566 | 3.69 |
| chr10 | 228 | 1.12 | 168 | 73.68% | 189 | 82.89% | 573 | 3.10 |
| chr11 | 227 | 1.12 | 144 | 63.44% | 165 | 72.69% | 834 | 3.89 |
| chr12 | 201 | 0.99 | 167 | 83.08% | 178 | 88.56% | 450 | 2.84 |
| all | 3140 | 1.29 | 2205 | 70.22% | 2457 | 78.25% | 728.5 | 3.58 |

| **Table S3**: Crossover overlapping rates of genomic regions and Monte Carlo simulation of crossover for genomic regions | | | | | | |
| --- | --- | --- | --- | --- | --- | --- |
| **Type** | **Overlapping crossover counts** | **Total number of  crossover (<5 kb)** | **Crossover overlapping rate** | **Mean simulated overlapping rate** | **Simulated standard deviation** | ***P*-value** |
| Intergenic*** | 1631 | 2457 | 66.38% | 85.30% | 3.07% | <2.20E-16 |
| Gene*** | 1403 | 2457 | 57.10% | 33.31% | 1.62% | <2.20E-16 |
| TSSsU1K*** | 599 | 2457 | 24.38% | 11.46% | 0.94% | <2.20E-16 |
| 5` UTR*** | 372 | 2457 | 15.14% | 5.24% | 0.78% | <2.20E-16 |
| Exon*** | 1273 | 2457 | 51.81% | 40.29% | 3.00% | <2.20E-16 |
| Intron*** | 838 | 2457 | 34.11% | 43.16% | 3.07% | <2.20E-16 |
| 3` UTR*** | 423 | 2457 | 17.22% | 5.80% | 0.81% | <2.20E-16 |
| TTSsD1K*** | 634 | 2457 | 25.80% | 11.47% | 0.95% | <2.20E-16 |
| The simulation was performed 10,000 times. | | | | | | |
| ***T-test (p<0.001) | | | | | | |

| **Table S4:** Crossover counts of each chromosome | | | | | | |
| --- | --- | --- | --- | --- | --- | --- |
| **Chromosome** | **Observed** | | | | | |
|  | **0** | **1** | **2** | **3** | **4** | **5** |
| chr01 | 22 | 71 | 72 | 29 | 8 | 1 |
| chr02 | 28 | 92 | 65 | 16 | 2 | 0 |
| chr03 | 26 | 79 | 67 | 29 | 2 | 0 |
| chr04 | 28 | 81 | 62 | 29 | 3 | 0 |
| chr05 | 46 | 83 | 63 | 10 | 1 | 0 |
| chr06 | 30 | 96 | 62 | 11 | 4 | 0 |
| chr07 | 51 | 91 | 51 | 7 | 3 | 0 |
| chr08 | 53 | 91 | 43 | 14 | 2 | 0 |
| chr09 | 26 | 81 | 83 | 10 | 2 | 1 |
| chr10 | 51 | 91 | 48 | 11 | 2 | 0 |
| chr11 | 45 | 98 | 51 | 9 | 0 | 0 |
| chr12 | 62 | 86 | 50 | 5 | 0 | 0 |
